# Lung Micrometastases Display ECM Depletion and Softening While Macrometastases Are 30-Fold Stiffer and Enriched in Fibronectin

**DOI:** 10.3390/cancers15082404

**Published:** 2023-04-21

**Authors:** Maria Narciso, África Martínez, Constança Júnior, Natalia Díaz-Valdivia, Anna Ulldemolins, Massimiliano Berardi, Kate Neal, Daniel Navajas, Ramon Farré, Jordi Alcaraz, Isaac Almendros, Núria Gavara

**Affiliations:** 1 Unit of Biophysics and Bioengineering, Faculty of Medicine and Health Sciences, University of Barcelona, 08036 Barcelona, Spain; mbarreto@ethz.ch (M.N.); africa.martinez@ub.edu (Á.M.); cjunior@ibecbarcelona.eu (C.J.); nataliadiazvaldivia@gmail.com (N.D.-V.); anna.ulldemolins@ub.edu (A.U.); neal@ub.edu (K.N.); dnavajas@ub.edu (D.N.); rfarre@ub.edu (R.F.);; 2 The Institute for Bioengineering of Catalonia (IBEC), The Barcelona Institute of Science and Technology (BIST), 08028 Barcelona, Spain; 3 LaserLab, Department of Physics and Astronomy, Vrije Universiteit Amsterdam, De Boelelaan 1081, 1081 HV Amsterdam, The Netherlands; massimiliano.berardi@optics11life.com; 4 Optics11, Hettenheuvelweg 37-39, 1101 BM Amsterdam, The Netherlands; 5 CIBER de Enfermedades Respiratorias (CIBERES), 08036 Madrid, Spain; 6 Institut d’Investigacions Biomèdiques August Pi i Sunyer (IDIBAPS), 08036 Barcelona, Spain; 7 Thoracic Oncology Unit, Hospital Clinic Barcelona, 08036 Barcelona, Spain

**Keywords:** lung metastases, melanoma, lung carcinoma, extracellular matrix, decellularization, atomic force microscopy, stiffness, fibronectin, basement membrane, nintedanib

## Abstract

**Simple Summary:**

This study examined the mechanical properties of the extracellular matrix (ECM) in lung metastases from two cancer models: lung carcinoma and melanoma. The ECM is the framework that holds tissues and organs together in the body. The researchers found that the ECM in the metastases was much denser and stiffer than healthy ECM. Fibronectin, a protein involved in cell adhesion, was overexpressed in both cancer models. Surprisingly, treatment with the anti-fibrotic drug nintedanib increased the stiffness of the tumor ECM and the amount of cell death (necrosis). The researchers suggest that targeting fibronectin and the mechanical properties of the tumor ECM could be a promising approach to cancer therapy and call for the development of new anti-fibrotic drugs to counteract abnormal ECM mechanics in metastases.

**Abstract:**

Mechanical changes in tumors have long been linked to increased malignancy and therapy resistance and attributed to mechanical changes in the tumor extracellular matrix (ECM). However, to the best of our knowledge, there have been no mechanical studies on decellularized tumors. Here, we studied the biochemical and mechanical progression of the tumor ECM in two models of lung metastases: lung carcinoma (CAR) and melanoma (MEL). We decellularized the metastatic lung sections, measured the micromechanics of the tumor ECM, and stained the sections for ECM proteins, proliferation, and cell death markers. The same methodology was applied to MEL mice treated with the clinically approved anti-fibrotic drug nintedanib. When compared to healthy ECM (~0.40 kPa), CAR and MEL lung macrometastases produced a highly dense and stiff ECM (1.79 ± 1.32 kPa, CAR and 6.39 ± 3.37 kPa, MEL). Fibronectin was overexpressed from the early stages (~118%) to developed macrometastases (~260%) in both models. Surprisingly, nintedanib caused a 4-fold increase in ECM-occupied tumor area (5.1 ± 1.6% to 18.6 ± 8.9%) and a 2-fold in-crease in ECM stiffness (6.39 ± 3.37 kPa to 12.35 ± 5.74 kPa). This increase in stiffness strongly correlated with an increase in necrosis, which reveals a potential link between tumor hypoxia and ECM deposition and stiffness. Our findings highlight fibronectin and tumor ECM mechanics as attractive targets in cancer therapy and support the need to identify new anti-fibrotic drugs to abrogate aberrant ECM mechanics in metastases.

## 1. Introduction

The extracellular matrix (ECM) is a three-dimensional network of macromolecules that provides structural and biochemical support to the embedded cells. Cells continuously interact with the ECM and rearrange, produce, and break down ECM components during physiological processes. During tumor development, the ECM undergoes complex structural changes that influence or even facilitate its progression, e.g., during invasion and metastasis formation [1,2,3,4]. In solid tumors, increased deposition of ECM components, a phenomenon known as desmoplasia, has been strongly linked to poor prognosis [5,6]. Increased protein deposition has been commonly linked to type I collagen (collagen I) [7,8,9] but can consist of other proteins such as elastin, hyaluronic acid, tenascin C, fibronectin, and laminin [10,11,12]. The presence (or absence) of certain ECM components is associated with differences in tumor development and progression. In fact, inhibition of fibronectin in human breast cancer cells led to a suppression of cancer growth in a metastatic mouse model [13]. In addition to biochemical modifications, structural rearrangements of the tumour microenvironment can influence cancer cell behavior as well, as seen in breast cancer matrices [14]. In the tumor stroma, cancer-associated fibroblasts (CAF’s) remodel tumor ECM and reorient collagen fibers perpendicularly to the tumor boundary, facilitating breast cancer cell invasion into the neighbouring tissues [14,15,16]. Clinical cases where collagen realignment has been detected have been linked to poorer prognosis and reduced therapeutic success [16]. 

This crosstalk between cells and the ECM is exerted by more than just the microenvironment components, as changes in the mechanics of the ECM provide different physical stimuli to embedded cells that significantly impact their development. Studies have determined that the stiffness of the ECM drives cell’s normal differentiation, migration, and proliferation [17], but also that aberrant ECM stiffness is associated with diseases such as pulmonary fibrosis [18,19] and malignancy [20,21,22]. For instance, it has been found that lysyl oxidase (LOX), which is an enzyme that crosslinks collagen and elastin, is overexpressed in many cancer tissues, such as gastric, colorectal, and breast cancer [23,24]. This overexpression increases tissue stiffness and promotes cancer invasion and progression [25]. Furthermore, stiffening of cancer tissue, attributed to an increase in ECM stiffening, has been linked to therapy resistance in pancreatic and hepatic cancer [7]. Additionally, several studies have shown that increasing matrix stiffness drives epithelial-to-mesenchymal transition (EMT) in breast and pancreatic cancer [7,26], leading to an overall increase in invasive behavior [27]. Finally, the stiffness of the matrix determines the levels of autophagy in smooth muscle cells, where stiffer substrates lead to higher levels of autophagy [28], a phenomenon often associated with resistance to treatment in cancer [29,30]. It is thus clear that changes in ECM mechanics—namely, its stiffening—lead to a variety of tumor cell responses linked to malignancy and drug resistance. 

Previous studies on a variety of cancers highlight an interesting contradiction: isolated cancer cells are softer than benign cells [31,32] but tumor samples (that is, the native tissue composed of cells and ECM) are found to be stiffer and more heterogeneous than healthy tissue [31,33,34]. This disparity stresses the need to study the ECM separately from cells and to assess its changes in the progression of malignancy. Indeed, the lack of studies on the mechanics of decellularized tumors has prevented the correlative assessment of tumor ECM stiffness with other tumor features, such as size, deposition of specific ECM components or the presence of necrosis. Furthermore, understanding ECM compositional and mechanical changes throughout each step of cancer progression will be useful for better understanding this process and for the development of targeted treatments. Notably, there is evidence that different ECM components play different roles in various tumor developmental stages. For example, although collagen is seemingly the primary component in the advanced tumor microenvironment, fibronectin is believed to play a key role in the formation of the pre-metastatic niche in pancreatic cancer [35]. 

Here, we focus on lung tumors, specifically metastases in the lungs, as the lung is the second most common site for metastatic cancer [36]. Furthermore, the mechanical features of the lung cell microenvironment are especially important because the pulmonary ECM is continuously subjected to cyclic stretching due to breathing. Our aim was to characterize the ECM progression of metastases in the lungs of mice, from both an endogenous source (Lewis lung carcinoma) and an exogenous source (melanoma). Additionally, we treated a group of mice with melanoma metastasis in the lungs with a clinically approved anti-fibrotic drug (nintedanib) to study its effects on tumor ECM. In all cases, tumors were decellularized and analyzed mechanically and compositionally, and these features were correlated with tumor size, presence of necrosis, and proliferation. We used this approach to show that the tumor is associated with a highly dense and stiff ECM, both in melanoma and lung carcinoma metastasis, and identified fibronectin as the key player in the tumor ECM, from the early to late stages of tumor development.

## 2. Materials and Methods

**Figure 1:**
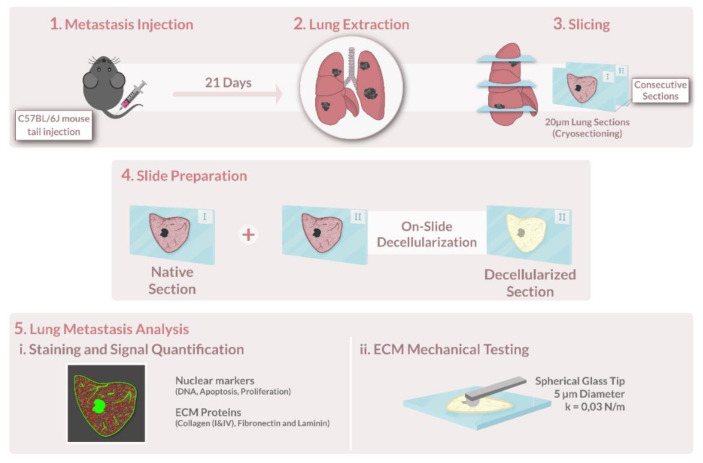
Schematic representation of the methodology followed in this work.

A schematic representation of the key steps in this methodology is shown in Figure 1.

### 2.1. Cell Culture 

Murine melanoma cells B16-F10 (ATCC, #CRL6475) and Lewis lung carcinoma (LCC1) cell line (ATCC, #CRL1642) were kept in high glucose Dulbecco’s Modified Eagle’s Medium (DMEM) (41966, GIBCO, Billings, MT, USA), buffered at pH 7.2–7.4, and supplemented with 10% fetal bovine serum (GIBCO, USA), penicillin–streptomycin solution (10^4^ U/mL and 10 mg/mL, respectively) (P4333, Sigma–Aldrich, St. Louis, MO, USA), and amphotericin B solution (250 mg/mL) (A2942, Sigma-Aldrich). Cell expansion was achieved by removing the whole medium, detaching the cells by rinsing the culture with 0.25% (*w*/*v*) Trypsin–EDTA (1× solution (25200, GIBCO, USA), and plating aliquots of the cell suspension in flasks incubated at 37 °C and 5% CO_2_. 

### 2.2. Lung Metastasis Assays

All animal studies in this work were approved by the Institutional Committee of Universitat de Barcelona and the Animal Experimentation Committee of regional authorities (Generalitat de Catalunya, OB 168/19 and 10972) following local and European regulations. 

This study was conducted on lungs from pathogen-free mice (8–12 weeks old; male; C57BL/6J; Charles River Laboratories, Saint Germain sur L’arbresle, France). All animals were housed in controlled cages and fed standard rodent chow (Panlab, Barcelona, Spain). Tap water was available ad libitum. Mice were injected intravenously either with 2 × 105 B16-F10 cells or 2.5 × 105 LCC1 cells (passages 4–5) in 100 μL of physiological saline on the tail vein. On day 21 post-injection, the animals were sacrificed, and the lungs were harvested and stored at −80 °C.

### 2.3. Tumor Decellularization

Decellularization of lung slices was performed as detailed in [37,38]. Briefly, acellular sections were produced by careful and consecutive washes and rinses of the lung section without detachment from the glass slide. Before decellularization, tissue sections were thawed at room temperature for 20 min and the edges of each slide were traced with a liquid repellent slide marker pen (Sigma-Aldrich) to keep liquid from spilling and minimize reagent usage. The OCT was removed by a 20-min incubation of PBS. Cells were lysed by 2 consecutive 10-min washes of ultra-purified water followed by 2 incubations of 15-min each of a mild detergent, sodium deoxycholate 2% (SD), to dissolve the cell membrane and detach cells from the matrix. After the removal of SD with 3 washes of PBS, a 20-min incubation of DNAse I solution (0.3 mg/mL, 5 mM MgCl_2_, 5 mM CaCl_2_ in 1 mM Tris-HCl) was performed to remove DNA remnants. The DNAse I was removed by 3 consecutive 5-min washes of PBS. After decellularization, samples were not frozen again and were immediately mechanically tested or stained.

### 2.4. Atomic Force Microscopy Measurements

The lung and lung tumor ECM were mechanically probed immediately after decellularization while submersed in PBS at room temperature (RT, 20 °C). The cantilevers used had a nominal spring constant (k) value of 0.03 N/m and a silicon oxide bead with a 5 µm diameter attached to its end (Novascan Technologies, IA, Chicago, IL, USA). Using a custom-built AFM system, the cantilever was moved by means of piezoactuators coupled to strain gauge sensors (Physik Instrumente, Karlsruhe, Germany) to measure the vertical displacement of the cantilever (z). The deflection of the cantilever (d) was measured with a four-quadrant photodiode (S4349, Hamamatsu, Japan). Prior to starting measurements, the slope of a deflection–displacement curve (d-z) was obtained from the indentation of a bare region of the glass slide. This curve was then used to calibrate the relationship between the photodiode signal and cantilever deflection—the deflection sensitivity. To check if the deflection sensitivity was calculated correctly, the bare region was indented again with this parameter updated. If this plot showed no indentation (perfectly perpendicular to the x axis) with a sharp contact point, then the calibration was considered successful, and it was taken as indicative of a clean undamaged tip.

With the visual assistance of the optical microscope, the tip was positioned macroscopically over the region of interest of the ECM. The deflection and displacement of the cantilever were recorded as the cantilever descended and contacted the sample surface at a constant speed of 15 µm/s, which is within the suitable velocity range for lung tissue indentation [39]. In each point, the sample was indented 5 consecutive times to reduce any measurement-to-measurement variability. In each region, each measuring point was separated by at least 20 µm. Between 3 and 5 points were measured in each region, and 3–5 regions were measured in the sample, for a total of 15–25 measurements per structure of interest (e.g., healthy lung ECM, tumor capsule, micrometastasis, etc.), unless the complete structure was measured in full in less than 15 indentations. The total duration of each AFM measurement on a given lung sample was ~ 3 h. At the end of the measurements, the probe was cleaned using helizyme (B. Braun, Germany) followed by ethanol 70% to remove any debris left on the probe.

### 2.5. Atomic Force Microscopy Data Analysis

Since the tip used in this work was spherical, we considered the Hertz contact model for a sphere indenting a semi-infinite half-space to be the most appropriate model, in line with previous works [18,40,41]:

(1) $$F=\frac{4E_{m}}{3(1-\vartheta^{2})}R^{1/2}\delta^{3/2},$$

where F is the force applied by the cantilever, δ is the sample indentation, R is the radius of the tip, ϑ the Poisson’s ratio of the sample (assumed to be 0.5), and $E_{m}$ is the microscale stiffness of the sample. To compute the model’s parameters, each force–deflection curve was fitted through a custom MATLAB code (MATLAB, The MathWorks Inc. Natick, MA, USA) by using a maximum of 2 µm of the indenting curve (due to the sample thickness of 20 µm and the tip radius of 2.5 µm). In this code, the Young’s modulus fitting was performed using the approaching curve and fitting the appropriate tip sample contact model to the force–indentation curve, as described in [41]. Indentations where the goodness of the fit (R^2^) was below 0.95, were discarded.

### 2.6. Dynamic Mechanical Analysis and Modelling

Viscoelastic properties can be quantified by a frequency-dependent complex shear modulus $E^{*}\left(f\right)$ [18,42,43]. This modulus can then be split between its real and imaginary components, as such:

(2) $$E^{*}\left(f\right)=E^{\prime }\left(f\right)+iE^{\prime \prime }(f),$$

where $E^{\prime }$ represents the storage modulus and $E^{\prime \prime }$, the loss modulus. The storage and loss modulus were measured by using the “Dynamic Mechanical Analysis” module of the Chiaro nanoindentation instrument (Optics11 Life, Amsterdam, The Netherlands) using the same setup as in [44]. The spherical tip used was similar to the one chosen for AFM measurements (glass, spring constant = 0.025 N/m and tip radius = 3 µm). In brief, for each probed location, the tip was set at an operating indentation of ≈1 µm. After a 30 s relaxation period, five 50 nm amplitude sine waves separated by 4 s relaxation periods were applied to the sample: 1 Hz (5 periods), 5 Hz (10 periods), 10 Hz (20 periods), 20 Hz (20 periods), and 50 Hz (20 periods). Measurements were performed at room temperature (20 °C). Each multifrequency acquisition had a duration of 120 s. A total of 6–10 locations were measured for each ECM region (i.e., healthy, ECM-rich). These values were averaged and then fitted in the complex plane to a two power-law model:

(3) $$E^{*}\left(f\right)=A{\left(if\right)}^{\alpha }+B{\left(if\right)}^{\beta },$$

where the first term of this model describes a low-frequency regime characterized by a weak exponent *α*, and the second term describes the high-frequency regime. As in previous works [18,42,45], we have fixed the parameter *β* to ¾ and all fits were performed using the online platform *fitteia* [46] (fitteia.org, accessed on 1 January 2019), which applies the non-linear least-squares minimization method. 

Based on the two-power law model, we defined the transition frequency ($f_{t}$) as the frequency where ${A(if_{t})}^{\alpha }={B(if_{t})}^{\beta }$, which represents the frequency at which the material; in this case, the ECM transitions from a storage modulus dominated domain (more solid-like) to a loss modulus dominated domain (more fluid-like), hence:

(4) $$f_{t}\;=\;exp\;(\frac{\ln\left(B\right)-ln(A)}{\alpha -\beta }).$$

### 2.7. Volumetric Tumor Determination

To perform non-invasive volumetric tumor determination, we assumed the three-dimensional shape of tumors conforms to a hemielipsoid, as per the current literature [47,48,49,50], defined by the following formula [48]:

(5) $$Volume=\frac{\pi }{6}\left(length\right)\cdot \left(width\right)\cdot (height)$$

The *length* and *width* of the tumor can be measured with great precision since they are directly observed, presenting a total error of 3% of the volume [51]. However, the *height* of the tumor presents a challenge, which can add 10.5% error to the volume value [48,52]. In order to reduce this error, an approach based solely on length and width measurements using the modified ellipsoidal formula was used [53]:

(6) $$Tumor\;volume=\frac{1}{2}(length\cdot {width}^{2}).$$

The length is the largest longitudinal diameter perpendicular to the largest transverse diameter or width. The measurements were taken with ImageJ digital image processing software [54] and were performed using either microscopic images of lung slices (micrometastasis) or a mixture of microscopic images and photos of the whole tumor (macrometastasis). 

### 2.8. Immunostaining of ECM Proteins, TUNEL and Ki67

Cellular and lung ECM sections were fixed with paraformaldehyde (PFA) 4% for 10 min at room temperature (RT). Samples were then blocked using a solution composed of 10% fetal bovine serum (FBS) and supplemented with 3% bovine serum albumin (BSA) for 1 h at RT and constant agitation (80 rpm). Lung sections were stained for ECM proteins: laminin, collagen type I, collagen type IV, and fibronectin. Cellular lung sections were additionally stained by Ki67, which was pre-conjugated with Alexa Fluor^TM^ 488 fluorophore (Ki67 Monoclonal Antibody (SolA15), Alexa Fluor™ 488, and eBioscience™, Thermo Fisher) to detect proliferation, and terminal deoxynucleotidyl transferase dUTP nick end labelling (TUNEL) to detect final stage apoptotic cells labelled using the Click-iT^TM^ Plus TUNEL Assay Kit (Thermo Fisher, Invitrogen), which was also pre-conjugated with Alexa Fluor^TM^ 488 fluorophore. Subsequently, primary antibodies against laminin (1:100, rabbit anti-laminin, Thermo Fisher), collagen type I (1:100, rabbit polyclonal to Collagen I, ab21286, Abcam), collagen type IV (1:100, Rabbit polyclonal to Collagen IV, ab6586, Abcam), and fibronectin (1:100, rabbit anti-fibronectin, ab2413, Abcam) were diluted in the same formulation as the blocking buffer and administered as to submerge the samples completely. Samples were incubated in primary antibody overnight, at 4 °C at constant agitation (80 rpm). To remove the primary antibody, sections were washed 3 times for 10 min with blocking solution at constant agitation (80 rpm). The secondary antibody (1:200, Goat Anti-Rabbit Cy3, ab97075, Abcam) was incubated diluted in the blocking buffer for 2 h at 37 °C and constant agitation (80 rpm). Three 10 min rinses with PBS were performed to wash out the unbound secondary antibodies. DNA was stained by incubation with Hoechst 33342 (NucBlue™ Live ReadyProbes™ Reagent, Thermo Fisher, Invitrogen) for 20 min at 80 rpm, followed by 3 5-min PBS washes to remove the solution. Hoechst staining’s concentration was 2 drops per ml, as per the manufacturer’s instructions. Finally, samples were mounted using Fluoromount mounting media (Invitrogen™ Fluoromount-G™ Mounting Medium, Thermo Fisher, invitrogen). 

### 2.9. Imaging and Image Processing

For full sample scanning and image stitching, epifluorescent imaging was performed with a Leica DMI 6000 epifluorescent microscope equipped with a Hammamatsu camera ORCA-Spark digital CMOS C11440-36U and a 5× objective. For higher resolution, fluorescent images of the tissue were acquired with a Leica SP5 inverted microscope equipped with a CCD camera (C9100, Hamamatsu Photonics K.K. Hamamatsu, Japan) and using a 10× and 20× Plan Fluor objective (Nikon).

**Figure 2:**
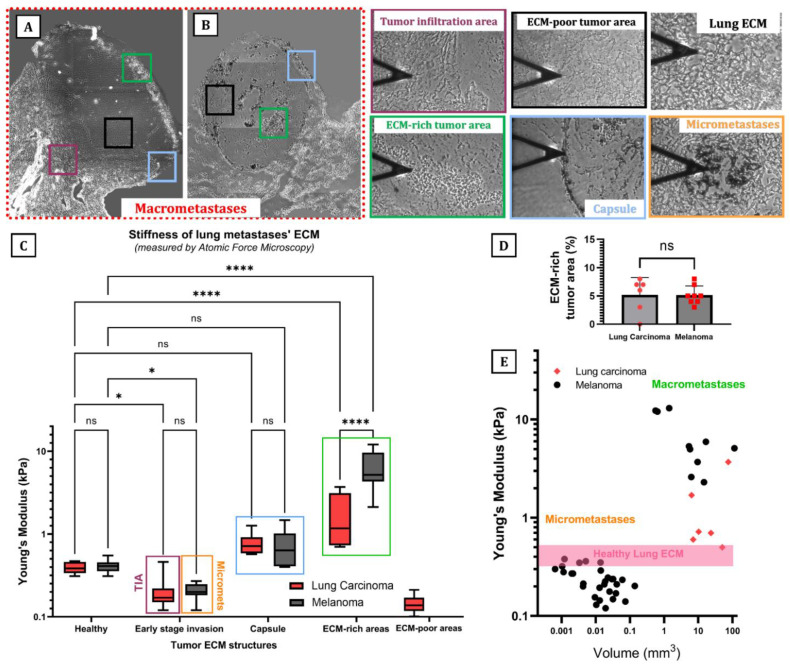
Structure and mechanics of the decellularized ECM of lung carcinoma (LC) and melanoma (MEL) lung metastases. (**A**,**B**) Lung carcinoma and melanoma decellularized metastases, respectively. Different ECM structures are marked with different coloured selections: green—ECM-rich regions; blue—ECM capsule; black—ECM-poor regions; purple—tumor-infiltration area (TIA); orange—micrometastases. (**C**) Young’s modulus (kPa) of each ECM structure of LC and MEL metastases, measured by atomic force microscopy. (**D**) ECM deposition measured by the area tumor area occupied by the ECM-rich areas, where the total tumor area was normalized to 100%. (**E**) Relationship between Young’s modulus (kPa) and estimated volume (mm^3^) of metastases. *p*-values were obtained using a two-way analysis of variance (ANOVA) with Tukey post hoc multiple comparisons. (ns, * *p*-value > 0.05, *p*-value < 0.05, ****, *p* < 0.0001).

**Figure 3:**
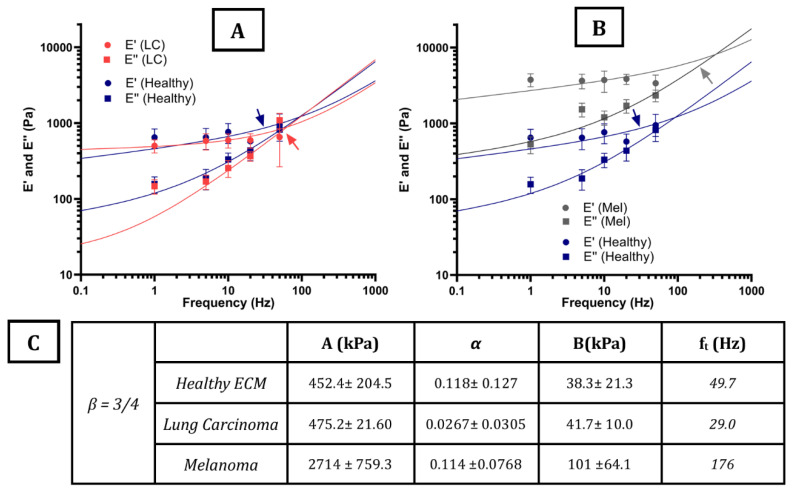
Frequency dependent loss (E′) and storage (E″) modulus as measured by dynamic mechanical analysis (DMA) of the ECM of healthy lung and the ECM-rich regions of (**A**) lung carcinoma (CAR) and (**B**) melanoma (MEL) lung macrometastasis. Solid lines represent the fits of the two-power law model. Arrows indicate the transition frequency. Fit parameters and transition frequency are detailed in table (**C**).

Brightness and contrast of phase contrast images of Figure 2 were improved for easier figure presentation. Since the same secondary antibody was used for staining all ECM proteins, the appearance of laminin, collagen I, and IV in fluorescent images was changed after image acquisition from red to yellow, blue, and green, respectively in Figure 2 for the same purpose. in Figure 3. 

Signal quantification was performed using ImageJ software. For ECM protein quantification, the “Freehand selections” tool was used to trace the edges of the area of interest (e.g., macrometastasis, micrometastasis, healthy area) using the phase-contrast image of the tumor ECM. Next, a mask was obtained based on this selection and applied to the corresponding fluorescent image (e.g., fibronectin, laminin, collagen). This selected area was then measured for mean intensity value and normalized using the mean intensity of the healthy ECM of the same lung section, as:

(7) $$Tumor\;protein\;intensity=\frac{Protein\;mean\;intensity\;in\;the\;tumor}{Protein\;mean\;intensity\;in\;the\;healthy\;region}\;\times \;100\%.$$

A similar procedure was followed for the computation of the area of the region of interest, but by normalizing with the area of the full tumor, as:

(8) $$Area\;of\;ROI\;=\;\frac{Area\;of\;ROI\;(pixels)}{Full\;tumor\;area\;(pixels)}\;\times \;100\%.$$

Such was the case for the computation of the necrotic, proliferative area and macrometastasis’ ECM production. 

To compute the coincidence of necrotic and proliferative areas with fibronectin and laminin tumor presence, the necrotic/proliferative positive areas in the FITC channel were traced and then masked. This mask was then applied to the fibronectin/laminin channel to determine the intensity of these proteins in the necrotic or proliferative tumor regions. This value would then be compared to the presence of the ECM proteins of the complete tumor area, as:

(9) $$Protein\;coincidence\;=\;\frac{Protein\;mean\;intensity\;in\;the\;ROI}{Protein\;mean\;intensity\;in\;the\;whole\;tumor}\;\times \;100\%.$$

A random distribution of the protein would yield results of approximately 100%, whereas a value of >100% means that the protein displays larger concentrations in the ROI than in the rest of the tumor. By always normalizing the results with regions of the same lung section, we decrease the influence of errors caused by irregular cryosections and immunostaining inconsistencies. 

Decellularization quantification was performed using the custom python algorithm described in detail in [55]. 

### 2.10. Nintedanib Treatment Assay

To simulate a clinical timeline, after 10 days post-metastatic cancer cell injection, mice previously injected with the murine melanoma cells B16-F10 started receiving treatment with Nintedanib (50 mg/kg, dissolved in PBS) by oral gavage. This treatment was administered daily for 11 days. On day 21 post-injection, the animals were sacrificed and the lungs were harvested and frozen at −80 °C.

### 2.11. Statistical Analysis

All data are mean ± SD. Normal and lognormal distributions of the datasets were tested using Shapiro–Wilk normality and lognormality tests. For comparison between two groups, since most groups followed a gaussian distribution, unpaired parametric student’s t-tests were used for all comparisons. Datasets with more than one group, on the other hand, followed a lognormal distribution. To achieve the Gaussian distribution necessary for further statistical analysis, datasets were converted to their logarithm and a two-way analysis of variance (ANOVA) with a Tukey post hoc multiple comparisons test was used. The relationship between variables was computed using Pearson’s correlations, as most variables followed a Gaussian distribution. To avoid clinically irrelevant correlations, we plotted relevant and/or significant correlations in Supplementary Figures S1 and S3. Differences were considered statistically significant at *p*-value < 0.05. Statistical analysis and graph generation were performed using GraphPad Prism (GraphPad software 9.1.0, Inc., San Diego, CA, USA).

## 3. Results

To study how metastasis from different origins—endogenous (lung) and exogenous (melanoma)—affect and remodel the lung ECM, we employed two well-established lung metastasis models [56,57] (lung carcinoma, LCC1 and melanoma, B16-F10). Lungs were harvested and cryosectioned but were not manipulated to remove metastasis from them; each cryosection contained both healthy lung ECM and tumorous ECM. This method was chosen because it resembles clinical conditions where the control tissue (“healthy tissue”) is taken from the biopsy of an adjacent area of the lung. To assess the lung and tumor ECM after the metastatic process, lung sections were decellularized without detaching from a glass slide, thus preserving the tumor’s internal structure [37,38]. 

### 3.1. Distinct ECM Structures Are Present throughout Tumor Development

The two tumor models produced macroscopically distinct tumors: lung carcinoma (CAR) metastases were rigid and colorless, while lung melanoma (MEL) metastases were viscous and black due to the melanin produced by the cancer cells. After decellularization, several ECM structures were clearly distinguishable microscopically (Figure 2A,B). Metastases were subdivided into two categories based on size and structure: micro- and macrometastases. Most macrometastases are surrounded by a thicker layer of ECM, which we refer to as the capsule. In melanoma macrometastasis, the tumor is strictly confined to the borders of the capsules. In CAR metastasis, capsules are thinner and at times non-existent. For that reason, CAR tumors often infiltrated the surrounding healthy tissue, and the ECM slowly transitioned from tumor ECM to “healthy” ECM—we call these transition regions, “tumor infiltration areas” (TIA)—with no clear boundary between healthy and tumor ECM, a phenomenon that was not present in melanoma metastasis. 

Within the tumor boundaries, tumor ECM had two distinct presentations: ECM-poor areas, where cancer cells had been previously predominantly located, and ECM-rich areas, which are areas of densely packed ECM that displayed a grape-like structure. In CAR tumors, the ECM-poor areas appear as a low-density meshwork of ECM proteins that covers most of the tumor area, transitions into neoplastic “tumor infiltration areas” (TIA) and finally to healthy lung ECM (Figure 2A). The ECM-rich regions of CAR tumors are located in the periphery and at the same location of the capsule (at times replacing it completely). This ECM distribution observed in lung carcinoma metastasis highly contrasts with the ECM of melanoma metastasis. Here, the ECM-poor areas within the capsule were mostly bare, with little to no ECM (Figure 2B), while the ECM-rich regions are more centrally located. Micrometastases were only present in the lungs of the melanoma metastasis’ model and their ECM consisted of what appears to be disorganized lung ECM, while the alveoli and blood vessels of the pre-metastatic site were at times still visible. 

To assess the area occupied by newly deposited ECM within the tumor, we computed ECM-rich areas as a percentage of the total tumor area. Even though they were located in different areas within the tumor, the percentage of ECM-rich areas in both CAR and MEL macrometastases was nearly identical (Figure 2D, 5.2 ± 3.1% and 5.1 ± 1.6%, respectively) which suggests that ECM production by the tumor does not depend on the tumor primary site. 

### 3.2. The Tumor ECM Is up to 15 Times Stiffer Than Healthy Lung ECM

Given that the tumor ECM structures appeared so different at the microscale, we hypothesized that they would also have different micromechanical properties. To that effect, we next sought to measure the Young’s modulus (E) of each ECM structure using Atomic Force Microscopy (AFM) and force–indentation curves (Figure 2C). 

As expected, the mean Young’s modulus ($E_{m}$) of the “healthy” lung ECM of mice injected with lung carcinoma and melanoma cells was nearly identical (0.39 ± 0.06 and 0.41 ± 0.07 kPa, respectively, *p* > 0.99). However, when compared with healthy ECM, the ECM of melanoma micrometastases showed a significant decrease in $E_{m}$ (0.20 ± 0.05 kPa, *p* = 0.018), which correlates with their visual presentation of a degraded lung ECM. The tumor-infiltrated areas (TIA) of lung carcinoma metastases showed an identical decrease in stiffness (0.21 ± 0.11 kPa, *p* = 0.036) when compared with melanoma micrometastasis (*p* > 0.99). A two-way ANOVA revealed a statistically significant interaction between the type of metastasis and tumor ECM structure (*p* < 0.0001). In addition, both the type of metastasis and ECM structure have a statistically significant effect on ECM stiffness (*p* = 0.0026 and *p* < 0.0001, respectively). Even though MEL metastatic cells travel to a new location while CAR invades the surrounding tissue, their identical ECM softening and similar visual presentations (i.e., overall lung ECM degradation) suggest that the early stages of tumor progression in both models have a common underlying mechanism for both endogenous and exogenous metastasis in the initial tumor invasion and progression stages. 

Even though the ECM capsules of both models had different visual presentations, the structures in both tumor types had similar microscale stiffnesses that were significantly higher than healthy lung ECM stiffness (0.78 ± 0.25 for CAR, *p* = 0.076 and 0.75 ± 0.41 for MEL, *p* = 0.31). This similarity also suggests a similar underlying mechanism when encapsulating the tumor, regardless of its cellular origin. Surprisingly, the ECM-rich regions of both models were more than 5- and 15-fold stiffer than the healthy ECM (1.79 ± 1.32 kPa for CAR and 6.39 ± 3.37 kPa for MEL, both *p* < 0.0001), a 9- and 32-fold increase, respectively, when compared to the softening measured in earlier tumor stages. This marked rise in stiffness suggests that this ECM is being newly produced by the tumor. However, the different stiffnesses and locations of the ECM-rich regions in CAR and MEL also suggest different underlying mechanisms for ECM production within the tumor. Lastly, the ECM-poor regions of the CAR metastasis were significantly softer than the healthy lung (0.15 ± 0.04 kPa, *p* = 0.0140), while the same regions in MEL metastasis were not measured due to a lack of measurable ECM.

### 3.3. ECM Stiffness Decreases with Tumor Size in micro- and Macrometastases 

Considering the varied visual presentation and size of the ECM structures found in our lung slices, we hypothesized that their heterogeneity likely captured the timeline of tumor progression (with smaller tumors reflecting earlier stages of tumor progression). With this in mind, we sought to correlate the stiffness of each tumor ECM with its volume (Equation (6)) (Figure 2E); thus, tumor volume was used as a surrogate for the development time. 

Regarding MEL metastasis development, our results show that the stiffness of smaller micrometastases is within the healthy ECM range (approximately 0.3 to 0.5 kPa) and, as they enlarge, AFM measurements showed that the ECM becomes progressively softer, indicating a degradation of the metastatic niche. At around 0.5 ${mm}^{3}$, micrometastasis become encapsulated, develop an ECM-rich region, and become macrometastatic. Surprisingly, this ECM-rich region also progressively decreased in stiffness with increasing tumor volume. Conversely, in CAR macrometastasis, there is no clear relationship between tumor volume and ECM stiffness. 

### 3.4. Lung Carcinoma Has Similar Viscoelasticity to Healthy ECM, While Melanoma Lung Metastasis Differs

To further characterize the lung metastases’ ECM—more specifically, its ECM-rich regions—we next set out to assess their viscoelastic behavior. Broadly, we used Dynamic Mechanical Analysis (DMA) to measure the ECM’s response to different probing frequencies. Even though the CAR stiffness values had been considerably higher than healthy ECM, the values for storage modulus $E^{\prime }$ and loss modulus $E^{\prime \prime }$ (Equation (2)) were practically identical (Figure 3A), where the storage modulus accounts for the stored elastic energy, while the loss modulus accounts for the dissipated energy. However, $E^{\prime }$ and $E^{\prime \prime }$ of MEL metastasis were an order of magnitude higher than in healthy lung ECM (Figure 3B), suggesting a sharp stiffening of the ECM, consistent with the quasi-static AFM results. 

These data were then fitted to a two-power law model (Equation (3)) (Figure 3C) in accordance to previously published works [18,45]. The coefficients A and B in this model can be interpreted as an index of stiffness at the low- and high-frequencies, respectively. Once again, these coefficients were similar in healthy lung ECM and ECM-rich regions of CAR metastasis. 

The ECM transition frequency ($f_{t}$) (Equation (4)) is the frequency at which the ECM transitions from a behavior closer to a solid to a more liquid behavior. This transition frequency was also similar in healthy lung ECM and CAR metastasis’ ECM (49.7 and 29.0 Hz, respectively) but was four times higher in melanoma metastasis (176 Hz). The α index of the power-law exponent is also related to the solid vs. liquid behavior of the sample, where a pure solid behavior is in the limit of *α* = 0 and fluidlike features *α* = 1. In our results, the *α* exponent is closer to 0 for all structures analyzed (*α* = 0.114 and 0.118, for healthy and MEL ECM, respectively), with a slight decrease of CAR ECM-rich regions (*α* = 0.027), indicating a more solid-like behavior in CAR tumor ECM and a more fluid-like behavior in MEL tumor ECM. 

Together, these DMA measurements and the Young’s modulus results measured by AFM suggest that most of the measured mechanical features of exogeneous metastasis (in this case, melanoma) greatly differ from its surrounding lung environment, while endogenous metastasis (lung carcinoma) display mechanical features that resemble more its initial metastatic niche.

### 3.5. Macrometastasis ECM Is Rich in Densely Packed Fibronectin but Has a Depleted Basement Membrane

**Figure 4:**
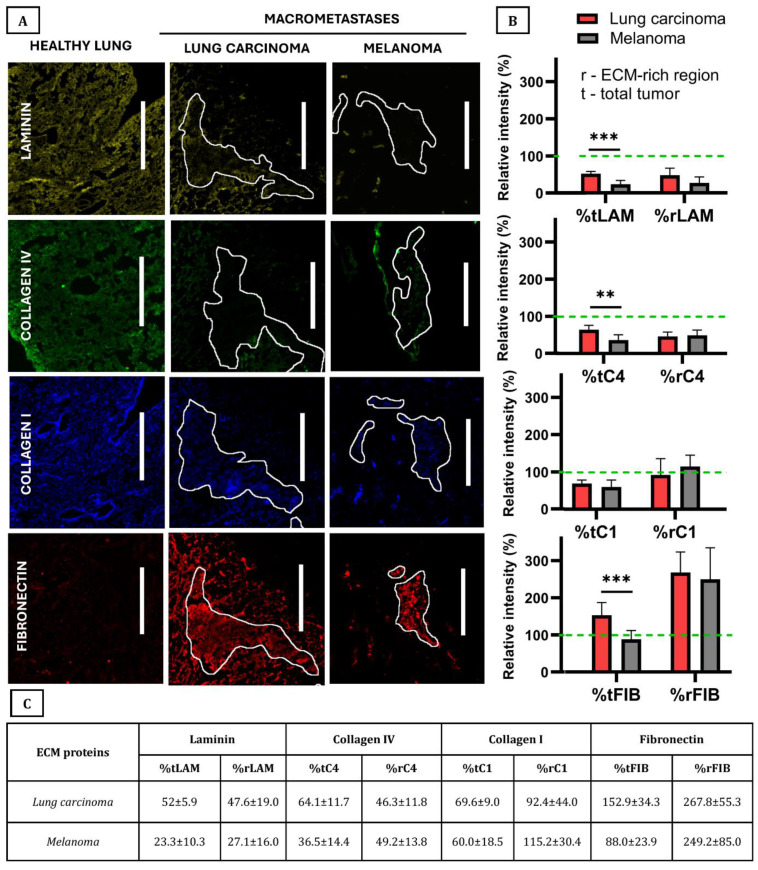
ECM protein staining and quantification of CAR and MEL macrometastases. (**A**) Representative fluorescent images of acellular tumor sections (20 µm) after immunostaining of laminin (yellow), collagen IV (green), collagen I (blue), and fibronectin (red). Healthy lung ECM is represented in the left column. ECM-rich areas (%r, white selections) were traced using a phase contrast image and then applied to the corresponding fluorescent image. (**B**,**C**) ECM proteins’ fluorescent signal quantification of the total tumor (%t) and tumor ECM-rich areas (%r). Healthy lung ECM signal was normalized to 100% (green line). Statistical analysis was performed using unpaired parametric student’s *t*-tests (**, *p*-value < 0.01, ***, *p*-value < 0.001). If nothing else is indicated, the relationship between two variables was found to be insignificant (*p* > 0.05). Scale bar = 500 µm.

After establishing the mechanical stiffening of the ECM in both endogenous and exogenous macrometastases, we hypothesized that the tumor was producing copious amounts of new ECM. To confirm this interpretation, we stained and quantified the presence of four ECM proteins that have established links to tumor development: two proteins from the interstitial ECM (collagen I and fibronectin) and two proteins from the basement membrane (collagen IV and laminin). To quantify these proteins, we used immunostaining to label each protein followed by fluorescence imaging and signal quantification (see representative images in Figure 4A. As a baseline, the fluorescence intensity of healthy ECM was measured, and this value was set to 100%. Tumor ECM proteins were quantified in two different areas: the total tumor area (%t_) and the ECM-rich areas (%r_) (Equations (7) and (8)). Overall, the basement membrane proteins, laminin, and collagen IV, were significantly reduced in the CAR and MEL metastases and in both the total tumor but especially in the ECM-rich regions. However, the presence of these proteins in the tumor was doubled in CAR tumors (laminin, 52 ± 5.9% and 23.3 ± 10.3% and collagen IV, 64.1 ± 11.7% and 36.5 ± 14.4%, in total CAR and MEL tumors, respectively), which reflects the ECM network that constituted the ECM-poor regions of CAR tumours but is absent in MEL tumors. In both tumor types, the levels of collagen I were similar: the total tumor levels were decreased (69.6 ± 9.0% and 60.0 ± 18.5%, for CAR and MEL, respectively) and the signal intensity in ECM-rich areas was roughly maintained (92.4 ± 44.0% and 115.2 ± 30.4%, for CAR and MEL, respectively) although slightly higher in melanoma metastases. Most importantly, fibronectin was significantly overexpressed in the ECM-rich regions of CAR and MEL metastases, reaching up to three times the baseline levels (267.8 ± 55.3% and 249.2 ± 85.0%, for CAR and MEL, respectively). For the total tumor levels of fibronectin, the MEL tumors levels were decreased (88.0 ± 23.9%), while the total fibronectin levels in CAR tumors were increased (152.9 ± 34.3%). 

### 3.6. ECM of Tumor Invasion Shows the Same Biochemical Signature in Lung Carcinoma and Melanoma, despite Different Phenotype

**Figure 5:**
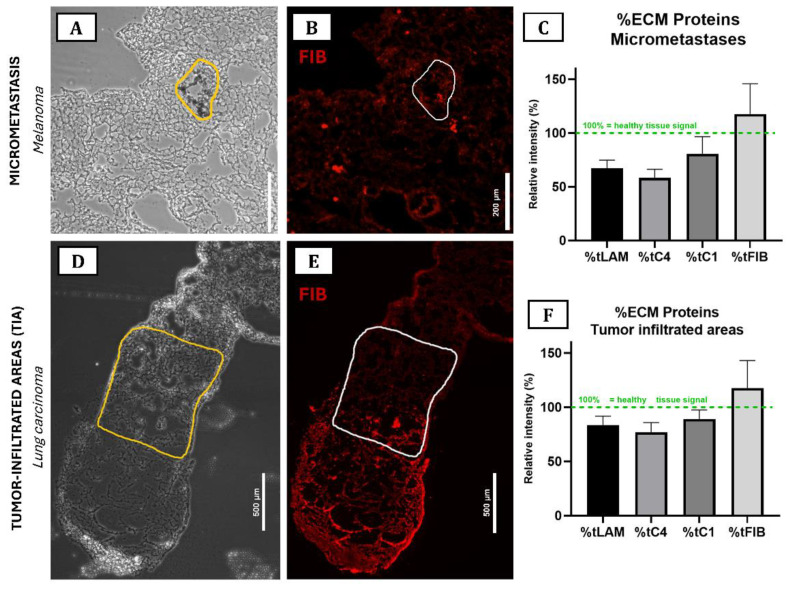
ECM protein staining and quantification of early-stage invasion areas in CAR metastases (tumor-infiltrated areas, TIA (**D**–**F**)) and MEL metastases (micrometastases (**A**–**C**)). Representative phase contrast (**A**,**D**) and corresponding fluorescent images (**B**,**E**) of acellular tumor sections (20 µm) after fibronectin immunostaining (red). The regions of interest were traced on the phase contrast images (yellow selection) and applied to the corresponding fluorescent image (white selection), and the signal was quantified. This quantification was performed for fibronectin, collagen I and IV, and laminin in micrometastases ECM (**C**) and TIA (**F**). The intensity of healthy lung ECM was normalized to 100% (green line).

The staining and quantification of ECM proteins in micrometastasis’ and TIA ECM were performed using the same method, but since these structures do not exhibit ECM-rich regions, only the total tumor ECM signals were computed. In micrometastases, all ECM proteins showed a decrease when compared to the healthy ECM baseline (67.3 ± 7.5% for laminin, 77.0 ± 8.9% for collagen IV and 89.3 ± 8.3% for collagen I) except for fibronectin, which showed a slight increase in mean signal (117.8 ± 28.2%). The same pattern was seen in TIA of lung carcinoma tumors, where there was a degradation of the overall ECM, although less prominent (83.4 ± 8.1% for laminin, 58.3 ± 8.1% for collagen IV and 80.7 ± 16% for collagen I)—except for fibronectin, which was overexpressed (117.8 ± 25.3%). This overall degradation of the ECM is consistent with our hypothesis that in the initial stages of the metastatic development, cancer cells degrade the pre-existing ECM, leading to ECM softening (Figure 2 and Figure 5).

Considering the presence of the ECM proteins in different locations and tumor stages, it is possible to assess a timeline for tumor ECM compositional dynamics. We hypothesize that when the MEL tumor initially metastasizes, it degrades the ECM as a whole, but especially the basement membrane (collagen IV and laminin) and stimulates the production of fibronectin, which is overexpressed already in micrometastasis. As the tumor grows, it continues to degrade the ECM while it expands in volume. At around 0.5 mm^3^, the tumor starts producing an ECM-rich area composed somewhat of collagen I and mainly fibronectin. The tumor progression in CAR metastasis is very similar, except that the degradation of the total tumor ECM is less prominent: although there is a reduction, the tumor forms a soft network of ECM proteins that could potentially be facilitating tumor infiltration to the surrounding tissues, as suggested by the transition from this ECM network into TIA and then into healthy ECM. As the tumor infiltrates additional healthy tissue, it again degrades its ECM but deposits new fibronectin.

### 3.7. Proliferation and Necrosis Do Not Depend on Metastasis Origin

**Figure 6:**
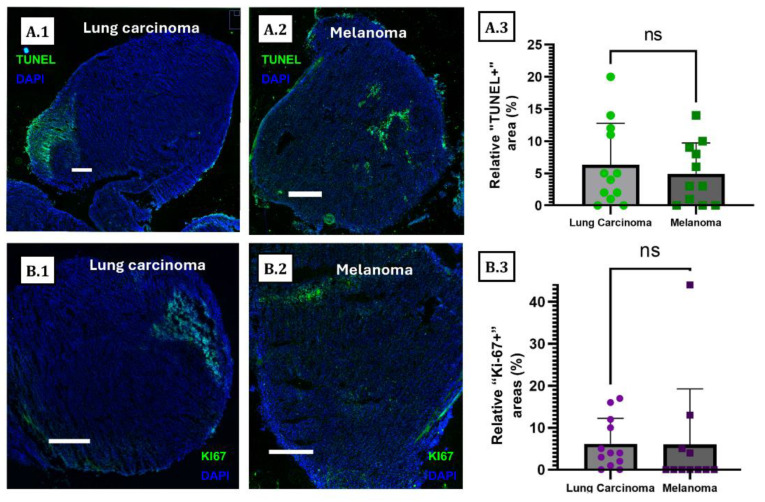
(**A.1**,**A.2**) Tumor necrosis (TUNEL) and (**B.1**,**B.2**) proliferation (Ki67) of CAR and MEL macrometastases. TUNEL+ and Ki67+ areas were selected and quantified by normalizing the total tumor area to 100% ((**A.3**,**B.3**), respectively). Scale bar = 500 µm.

After establishing the differences in ECM composition and mechanics between the two tumor models, our next step was to assess if other cellular tumor characteristics such as proliferation and necrosis were dependent on the metastasis site or origin. For this purpose, consecutive native slices of the tumors were stained for necrotic (TUNEL) and cell proliferation (Ki67) markers. Images were quantified by selecting the tumor regions that were positive for these stainings, computing their area and normalizing to a percentage, where 100% equals the total tumor area (Figure 6A.3,B.3). As with the location of the ECM-rich areas, the location of the TUNEL+ areas in CAR and MEL tumors differed in the same way: in CAR tumors, TUNEL+ regions were located peripherally, while in MEL tumors, TUNEL+ regions were more centrally located. However, the number of TUNEL+ regions was similar in both types of metastases (Figure 6A.3, 6.3 ± 6.4% and 4.9 ± 4.8%, for CAR and MEL, respectively), albeit slightly higher for CAR tumors. Regarding Ki67 staining for cell proliferation (Figure 6B.1–B.3), not all tumors were positive for Ki67 markers, especially in MEL tumors. However, the mean “Ki67+” areas were virtually the same in both models (6.2 ± 6.1% and 6.0 ± 13.2%, for CAR and MEL, respectively). These findings suggest that tumor proliferation and necrosis are dependent on the metastatic niche’s location and not on the origin of the metastasis. Furthermore, the similar location within the tumor of necrotic areas and the ECM-rich regions in Figure 2A,B suggests a link between tumor angiogenesis and tumor ECM deposition. 

After assessing the tumor size, composition, mechanics, necrosis, proliferation, and ECM content, we set out to find relevant correlations between these parameters via a Pearson matrix correlation for each type of metastasis: CAR and MEL (Supplementary Figure S1). As expected, stiffer ECM-rich areas are (weakly) correlated with an overall increase in the abundance of ECM proteins. However, this is not the case for collagen I in CAR macrometastases, where the relationship is the opposite. In MEL, the area occupied by ECM-rich regions decreased with increasing tumor diameter, a relationship absent in lung carcinoma. In MEL metastases, it was possible to track the presence of ECM proteins throughout tumor progression (Supplementary Figure S1F), since the tumor’s diameter could be measured from the early invasion stages (micrometastases). This analysis clearly showed that basement membrane proteins (laminin and collagen IV) were continuously degraded, from the micro- to the macrometastatic stage, whereas fibronectin levels increased with tumor growth. However, collagen I levels are approximately maintained in the different tumor stages. 

### 3.8. Nintedanib Causes a Two-Fold Increase in ECM Stiffness and Four-Fold Increase in ECM Deposition 

**Figure 7:**
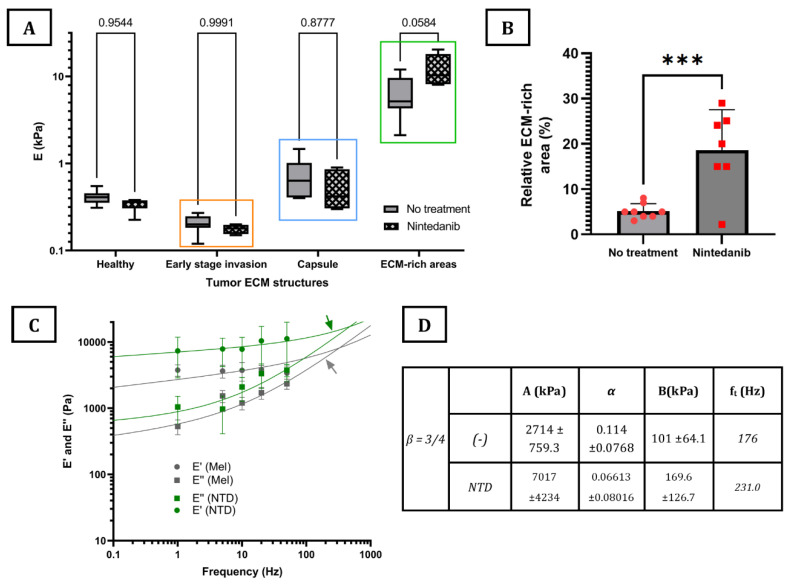
Nintedanib effects on structure and microscale mechanics of melanoma lung metastases. (**A**) Atomic force microscopy (AFM) measurements of the Young’s modulus (kPa) of each ECM structure of MEL metastases that either received no treatment or treatment with NTD. *p*-values were obtained using a two-way analysis of variance (ANOVA) with a Tukey post hoc multiple comparisons. (**B**) ECM deposition of ECM-rich regions of MEL macrometastases with and without NTD treatment. Measured by the tumor area occupied by the ECM-rich areas, where the total tumor area was normalized to 100%. Statistical analysis was performed using unpaired parametric student’s *t*-tests (***, *p*-value < 0.001). (**C**) Frequency dependent loss (E′) and storage (E″) modulus as measured by dynamic mechanical analysis (DMA) of the ECM of ECM-rich regions of MEL lung macrometastasis in NTD treated and non-treated mice. Solid lines represent the fits of the two-power law model. Arrows indicate the transition frequency. Fit parameters and transition frequency are detailed in table (**D**).

Due to their well-established role in modulating the tumor microenvironment and tumour ECM turnover, we also suspected that cancer associated fibroblasts were involved in the production of the tumor ECM. For these reasons, we chose to use Nintedanib (NTD) (Figure 7), which was originally developed for oncological applications due to its anti-angiogenic features, but it is believed to also modulate fibroblast proliferation and activation [58]. NTD is an oral medication used for the treatment of idiopathic pulmonary fibrosis and non-small cell lung cancer. We chose to use NTD alongside the melanoma lung metastasis model only, due to this cancer model’s more prominent mechanical changes, structure, and the presence of micrometastases as well as a proof-of-concept for future ECM-modulating studies. The same sample preparation, cryosectioning, and decellularization protocols previously described were followed for this treatment model. 

NTD treatment did not change the previously described structures of the MEL ECM in macrometastasis, since the surrounding ECM capsule, the ECM-rich regions within the capsule, the ECM-poor regions (which were mostly bare), and the micrometastases were all present in NTD treated mice. We performed AFM and DMA on NTD treated tumors (Figure 7) and, as expected, NTD treatment softened many of the ECM structures. “Healthy” lung ECM was softened after treatment (0.34 ± 0.05 kPa and 0.41 ± 0.07 kPa, with and without NTD treatment, *p* = 0.9544); the capsule (0.55 ± 0.29 kPa, *p* = 0.8777) and the micrometastases (0.18 ± 0.02, *p* = 9991) were also softened with NTD treatment, but not significantly. However, this softening was not observed inside the tumor since NTD caused a 2-fold increase in the stiffness of the ECM-rich regions of the macrometastasis when compared to mice that received no treatment (6.39 ± 3.4 kPa and 12.35 ± 5.74 kPa, respectively, *p* = 0.0584). Most surprisingly, not only was the NTD treated tumor ECM much stiffer, but the amount of ECM-rich occupied areas in macrometastases almost quadrupled (5.1 ± 1.6% to 18.6 ± 8.9%, *p* = 0.0010). 

The same DMA protocol and the two power-law model fit described previously was applied to NTD treated ECM-rich regions of the melanoma macrometastasis. NTD treatment caused a further increase in the values for storage ($E^{\prime }$) and loss modulus ($E^{\prime \prime }$), in-line with the stiffness increase already measured with AFM. In addition, NTD treatment also resulted in the increase in transition frequency from 176 to 231 Hz, indicating solidification of the ECM (Figure 7C,D). However, the low frequency α index of NTD-treated ECM-rich regions was half of non-treated melanoma ECM (*α* = 0.066 and 0.114, respectively). 

### 3.9. Nintedanib Increases Tumor Necrosis and Proliferation

**Figure 8:**
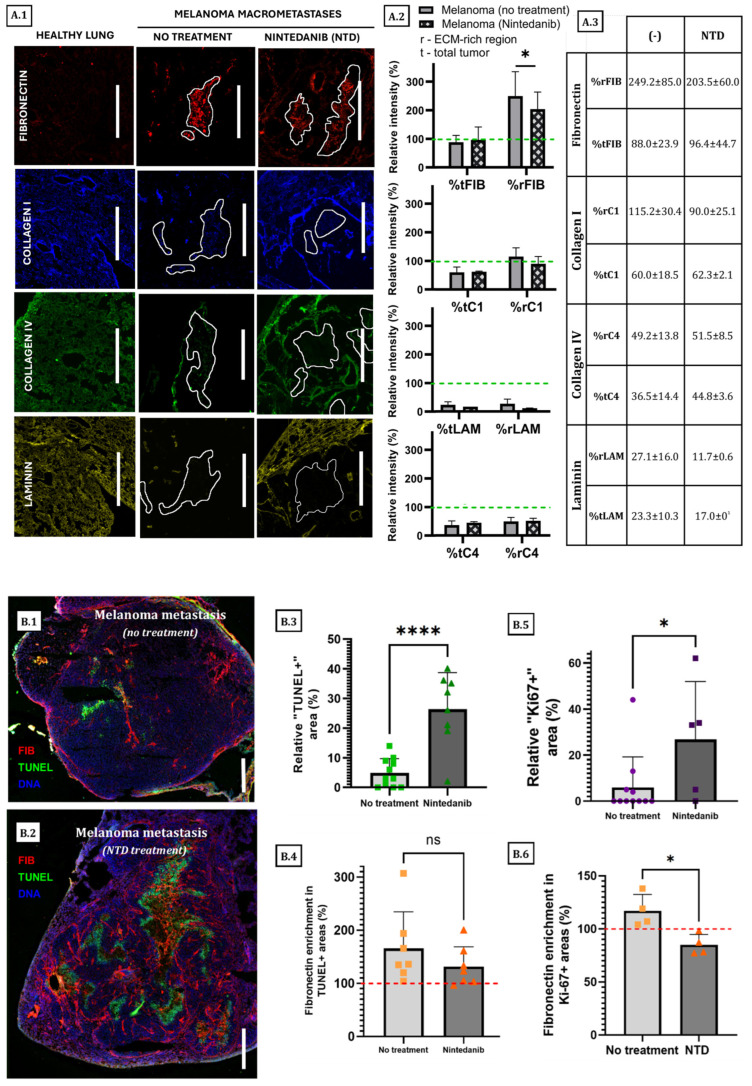
Effects of Nintedanib on the ECM composition, proliferation, and tumor necrosis of MEL macrometastases. (**A.1**) Representative fluorescent images of acellular tumor sections (20 µm) after immunostaining of laminin (yellow), collagen IV (green), collagen I (blue), and fibronectin (red). Healthy lung ECM is represented in the left column. ECM-rich areas (%r, white selections) were traced using a phase contrast image and then applied to the corresponding fluorescent image. (**A.2**,**A.3**) ECM proteins’ fluorescent signal quantification of the total tumor (%t) and tumor ECM-rich areas (%r). Healthy lung ECM signal was normalized to 100% (green line). (**B**) Effect of Nintedanib treatment on tumor necrosis (TUNEL) (**B.1**–**B.4**) and proliferation (Ki67) (**B.5**,**B.6**). TUNEL+ and Ki67+ areas were quantified in (**B.3**,**B.5**), respectively. (**B.4**,**B.6**) Fibronectin enrichment in (**B.4**) TUNEL+ areas and (**B.6**) Ki67+ areas 100% corresponds to the total tumor measurement. Statistical analysis was performed using unpaired parametric student’s *t*-tests (ns, *p*-value > 0.05 *, *p*-value < 0.05, ****, *p* < 0.0001). Scale bar = 500 µm.

After determining that NTD clearly changed the mechanical properties of the tumor ECM, the next step was to assess changes in necrosis, proliferation, and ECM composition. The same ECM quantification method was applied to tumors treated with NTD (Figure 8, Supplementary Figure S2). Even though the NTD treated tumor ECM was more abundant and stiffer (Figure 7A,B), the composition of this tumor ECM did not differ much from that of non-treated melanoma ECM, since there were no significant changes between the composition of both tumor types (Figure 8A.1–A.3).

On the other hand, NTD treatment of melanoma metastasis caused a significant and prominent increase in the necrotic areas of the tumors, from 6.3 ± 6.4% to 26.4 ± 12.3% of the total tumor area (Figure 8B.1–B.3, *p* < 0.0001), which was consistent with the NTD’s anti-angiogenic mode of action. There was also a significant increase in proliferation in the tumors (from 6.0 ± 13.2% to 26.8 ± 25.1 of total tumor area, *p* = 0.0445) (Figure 8B.5). To study whether tumor necrosis and proliferation were connected to fibronectin deposition, we computed the fibronectin enrichment of the TUNEL+ and Ki67+ areas compared to the average fibronectin content of the whole tumor (Equation (9)). Results equal to 100% indicate that the distribution of the fibronectin in the area of interest is the same as the distribution in the rest of the tumor; whereas results higher/lower than 100% mean that the fibronectin is enriched/decreased in this area of the tumor compared to the average distribution throughout the tumor, respectively. Computing these parameters showed that there was substantial fibronectin enrichment in necrotic areas in tumors treated and not treated with NTD (173.7 ± 72.14% and 131.6 ± 37.4%), while this enrichment was only found in non-treated metastasis in Ki67+ areas (117 ± 15.43% and 85 ± 9.8%, for non-treated and treated metastasis, respectively, *p* = 0.0127) (Figure 8B.4,B.6).

### 3.10. Necrosis Positively Correlates with ECM Deposition and Stiffening, in Mice Treated with Nintedanib

**Figure 9:**
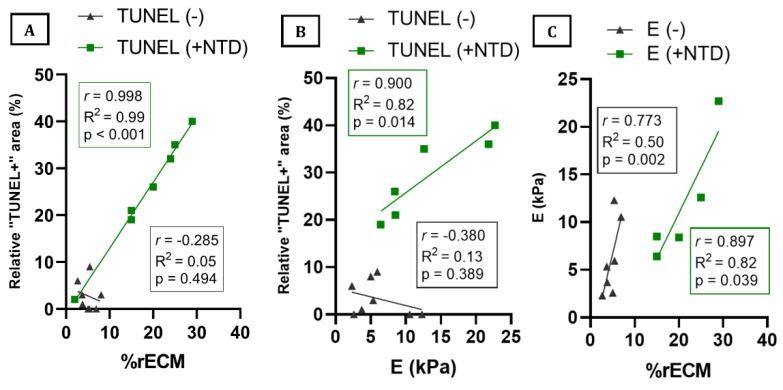
Correlation analysis of ECM-rich area (%rECM), tumor necrosis (TUNEL+), and the stiffness of the ECM-rich areas (E(kPa)) in melanoma macrometastases that received no treatment (triangles, −) and received nintedanib treatment (squares, +NTD) for 10 days after initial cancer cell injection. (**A**) Correlation analysis between tumor necrosis and ECM production. (**B**) Correlation analysis between tumor necrosis and ECM stiffness of ECM-rich areas. (**C**) Correlation analysis between ECM-rich stiffness and relative tumor area. *p*-values (*p*) and correlation coefficient (*r*) were obtained by computing the Pearson correlation.

We produced a Pearson correlation matrix using treated and non-treated tumor features (Supplementary Figure S3A) to identify the significant relationships (Supplementary Figure S3B–E and Figure 9). 

As NTD is anti-angiogenic and anti-fibrotic, we were especially interested in studying the relationship between melanoma tumor necrosis (caused by insufficient tumor vascularization), ECM deposition, and tumor ECM stiffness. After the suppression of tumor vascularization via NTD treatment, a strong positive relationship between the amount of tumor necrosis and the amount of ECM deposition within the tumor (r = 0.998, *p* < 0.001) is clearly established (Figure 9A), which is not observed in non-treated tumors (r = −0.285, *p* = 0.494). This likely means that, in NTD treated mice, the more necrotic the tumor is, the more ECM has been produced. Tumor necrosis and tumor ECM stiffness show a similar association (Figure 9B): the more necrotic the tumor is, the stiffer its ECM-rich areas are (r = 0.900, *p* = 0.014)—a relationship that is not observed in non-treated tumors (r = −0.380, *p* = 0.389). However, both tumor models show a positive relationship between ECM stiffness and ECM deposition (Figure 9C), meaning that the more tumor ECM is produced, the stiffer it is (r = 0.773, *p* = 0.002; r = 0.897, *p* = 0.039 for NTD-treated and non-treated tumors, respectively). These relationships suggest an underlying mechanism that intimately connects tumor angiogenesis, tumor ECM deposition, and stiffness.

## 4. Discussion

In this study, we characterized the ECM of lung metastases by decellularizing tumorous sections from two different metastatic models: melanoma lung metastases (B16-F10) and Lewis lung carcinoma metastases (LLC1). We show that the tumor ECM is not as disorganized and unregulated as previously thought [59,60], and that desmoplasia is not restricted to the tumor surroundings [5,61]. Upon decellularization, it became clear that different ECM structures were likely formed by different mechanisms, and not by random deposition or degradation of the pre-metastatic ECM. In contrast to previous studies, our work aimed to assess the mechanics of the ECM throughout metastasis progression and with different primary site origins. We were able to show that ECM mechanics can reflect the underlying malignant mechanisms and evolve alongside tumor progression, from the initial stages of tumor invasion to macrometastases. This study also linked ECM mechanical changes to changes in ECM composition, such as softening caused by basement membrane degradation and the identification of fibronectin as a key ECM protein displaying deposition changes in every stage of lung metastasis progression. When comparing lung carcinoma and melanoma lung metastases, we found substantially different ECM phenotypes and mechanics, although proliferation, cell death, and ECM composition were the same. Nintedanib treatment of melanoma metastases further reveals the intimate link between angiogenesis and ECM deposition, which will aid in the development of new tumor microenvironment-targeting therapeutics.

One of the main objectives of this study was to compare the tumor ECM of two different metastatic origins but on the same target-organ, according to the “seed and soil” hypothesis [62]. Further developments of this hypothesis have postulated whether the seeds recreate their own microenvironment into the new soil or whether they depend on the target organs’ features to establish a compatible metastatic niche after their arrival [60]. In our study, there was evidence for both. The mechanical features of the tumor ECM support the theory that different origins produce different tumor environments by attempting to recreate their original microenvironment. In fact, most mechanical parameters measured in lung carcinoma, such as stiffness, transition frequency, and storage and loss modulus, were much closer and sometimes equal to that of the healthy lung ECM, the primary site of CAR metastases. In contrast, the ECM of melanoma metastases had strikingly different mechanics than the surrounding healthy tissues (Figure 2 and Figure 3). This evidence suggests that the mechanical properties of the ECM are dependent on the primary site of secondary lesions. However, even though the locations of the ECM-rich regions within the tumor were different, their composition was roughly the same regardless of the metastatic origin (Figure 4). In fact, proliferation, cell death markers, and the amount of ECM deposition were very similar in both mouse models (Figure 6). These similarities could be attributed to an influence of the target organ environment (e.g., the “soil”) on tumorigenesis rather than the primary organ site (e.g., the “seed”). 

Lung carcinoma and melanoma lung metastases also exhibited different invasion phenotypes: melanoma metastasized to a different location, giving rise to micrometastases, while lung carcinoma invaded nearby tissues resulting in tumor-infiltrated areas (TIAs). The difference in these presentations could be due to differences in tumor encapsulation, since only melanoma metastases were fully encapsulated and thus could not easily expand and invade nearby tissues. In fact, it has been suggested that incomplete tumor capsules constitute a risk factor for invasion in hepatocellular carcinomas [63]. Although different in their presentations, the ECM of these two early-stage invasion regions showed the same softening and a very similar composition (Figure 1D and Figure 5). In both cases, there was a clear degradation of the basement membrane components (laminin and collagen IV), which results from cancer cell invasion of new tissues through the basement membrane and has been observed in many cancers, such as ovarian and lung carcinomas [64,65,66]. Degradation of the basement membrane is achieved by MMPs and facilitates the invasion of metastatic cancer cells, but also allows for the growth of new blood vessels during tumor progression [67]. This degradation presumably led to progressive softening of the ECM with increased tumor size in micrometastases (Figure 2), which indicates the destabilization of the ECM as the tumor invades the tissue. Surprisingly, however, both models of early-stage tumor invasion showed an 18% increase in fibronectin. In the case of the melanoma micrometastases, fibronectin deposition could be overexpressed to generate a more suitable “soil” for metastases, which is in line with reports that fibronectin promotes the settling of circulating cancer cells and has many pro-metastatic and tumorigenic qualities [13,68]. Recent work has shown that some tumors can prime “pre-metastatic niches” in target-organs before cancer cells leave the primary tumor site. In breast cancer [69], researchers have found that this preparation occurs via the deployment of vesicles that will promote the production of growth factors and ECM proteins to create a welcoming environment for the establishment of a new tumor, namely, fibronectin to promote tumor cell engraftment, growth, and the recruitment of MMPs to degrade the basement membrane [60], which is in line with our results. 

Decellularization of macrometastases also revealed a dense ECM structure inside the tumor, which we called the ECM-rich region. In melanoma metastases, the stiffness measured in this structure could reach up to ~40 kPa, which is 100 times stiffer than healthy ECM, whereas in the lung carcinoma model ECM stiffness reached a maximum of ~10 kPa. In both metastatic mouse models, this increase in stiffness can be partially explained by the stark increase in fibronectin when comparing healthy and macrometastatic ECM (Figure 4). However, the correlation between stiffness and fibronectin presence, although positive, was weak and non-significant (Supplementary Figure S1C,E). This observation indicates that the stiffness of the ECM cannot be solely determined by the number of ECM components, which is corroborated by the fact that the CAR ECM, although significantly softer than melanoma ECM, expressed more fibronectin, collagen type IV, and laminin than melanoma metastases. The increase in stiffness could then be due to an ECM protein that we have not tested, but we propose that it may be due to the well-reported overexpression of LOX in intra-tumoral environments [23,24,70], which covalently crosslinks collagen thereby increasing tissue stiffness. LOX is induced by hypoxic conditions in the tumor [71,72] and its pro-tumorigenic role has been well described [73,74]. Thus, hypoxia in tumors may induce ECM stiffening, which, in turn, promotes angiogenesis by stimulating the release of growth factors such as vascular endothelial growth factor (VEGF) [75]. In fact, when analyzing ECM stiffness and development in relation to tumor volume, we can see that the first ECM-rich regions were measured in ~1 mm^3^ volume macrometastases, which is consistent with the reported volume at which tumors switch from relying on pre-existing blood supply to a angiogenic phenotype, of approximately 1–2 mm^3^ [76,77,78]. 

This hypothesis was further corroborated by the results obtained after nintedanib treatment. Here, VEGF receptor tyrosine kinases are inhibited (along with other major angiogenic tyrosine kinase receptors), which inhibits tumor angiogenesis and thus leads to a sharp increase in cell death (Figure 8B.3) compared to non-treated tumors, which is consistent with previous studies using nintedanib [79]. Since these hypoxic conditions are not ameliorated due to the lack of new blood vessels, LOX is likely continuously secreted into the tumor as hypoxia increases, which could explain the doubling in ECM stiffness we reported after nintedanib treatment. This hypoxia–LOX–stiffness relationship was further corroborated by the strong positive correlation between cell death and tumor ECM stiffness (Figure 9B), which was only established after NTD treatment. The link between cell death and ECM deposition is further clarified in Figure 9A since the administration of an anti-angiogenic drug causes a linear relationship between tumor necrosis and ECM deposition, where the more necrotic a tumor is the more ECM is produced. This relationship hints at the modulation of intra-tumoral ECM deposition as a tool to hinder tumor necrosis and hypoxia. 

The relative locations of the ECM-rich regions within the tumor also indicated a connection between ECM deposition and tumor necrosis, as they largely overlapped. To verify this hypothesis, we computed the fibronectin enrichment of the TUNEL positive areas, since fibronectin is the main component of ECM-rich areas (which we have equated to ECM deposition). We found that, in fact, TUNEL positive areas had much more fibronectin than the rest of the tumor. This evidence implies that tumor hypoxia and cell death are connected not only to ECM deposition in general, but also to fibronectin deposition specifically, which can also be explained by LOX overexpression. In fact, Wu and colleagues showed that LOX was not only upregulated in higher stiffness substrates in an in vitro model; it also promoted the production of large quantities of fibronectin by resident fibroblasts [80,81]. Notably, fibronectin expression is also linked to an increase in angiogenesis in the tumor, since it facilitates endothelial cell survival, a crucial step in the formation of new blood vessels [59], which could explain its overexpression in the necrotic core. 

Although many studies have singled out collagen I as the main player in tumor ECM [9,11,12,66], in our study, fibronectin was the main protein overexpressed throughout the different stages of lung metastases progression, especially in macrometastases. Recent studies have begun to identify fibronectin as an important component of the tumor ECM. In a 2021 study, Ghura and colleagues showed that inhibiting circulation of fibronectin in breast cancer and melanoma mice models led to significant suppression of tumor growth, while inhibition of collagen type I led to no such changes [13]. Based on these findings, as well as the ones described in this study, we propose that targeting tumor fibronectin could not only suppress cancer growth but also prevent the formation of the pre-metastatic niche and consequent metastatic invasion, opening up a wide range of therapeutic possibilities. Future studies should also delve deeper into the influence of fibronectin on tumor progression, namely, by identifying the specific fibronectin isoforms produced in the tumor, as well as other relevant proteins that have not been covered in this work, such as tenascin-C and versican [82,83].

NTD is one of the currently approved therapeutic options for lung cancer, specifically non-small cell lung cancer. Although NTD has been progressively recognized as an invaluable anti-fibrotic drug, especially for idiopathic pulmonary fibrosis, its anti-fibrotic actions have not been fully elucidated. As expected, after NTD treatment, there was generalized softening of most ECM structures, although not significant (Figure 7). Surprisingly, NTD caused a 4-fold increase in ECM deposition and 2-fold increase in ECM stiffness in mice with melanoma metastases. Upon closer observation, the ECM structures that softened were those that were not directly produced by the tumor components, but rather derived from pre-existing lung ECM (the healthy ECM, the ECM capsule as a result of the tumor expansion, and the micrometastatic ECM, resulting from the degradation of the healthy ECM after cancer cell invasion). This evidence suggests that the anti-fibrotic activity of NTD did not produce an effect inside the tumor but only on its surroundings and structures derived from lung ECM. Another explanation for the reported results is that the effects produced by the anti-angiogenic features of NTD override the supposed anti-fibrotic effects, namely, the sharp increase in cell death as a result of poor tumor vasculature and hypoxia, which strongly correlated with increased tumor ECM stiffening and deposition (Figure 9). Previous studies have also shown limited anti-fibrotic action after NTD administration. Namely, some studies have shown no alteration in fibrosis markers or collagen I expression in patients with IPF (idiopathic pulmonary fibrosis) [84,85] after NTD administration. In a brain and breast cancer metastases model, NTD also showed no anti-ECM effects [86,87]. Collectively, our results and those of others suggest that further investigation into the effect of NTD on the ECM is required to fully understand the effects of this drug in the tumor microenvironment. 

By measuring the mechanical characteristics of the tumor ECM, AFM measurements of the tissue can be applied in diagnostics by distinguishing healthy from malignant tissue or by determining the invasive potential of a tumor. Plodinec et al. (2012) used AFM to measure the stiffness of human breast cancer biopsies. The study concluded that the mechanical properties of the tissue can be used as a distinct mechanical fingerprint of cancer-related changes, differentiating between normal, benign, and invasive tumours [31]. If these findings can be applied to other types of cancers at different stages, nanomechanical signatures can have high diagnostic potential. In the case of lung metastases, if the mechanics of the metastatic ECM are consistent with the primary tumor, clinical mechanical measurements could aid in the identification of the primary site, especially in cases where histological assessment is insufficient (e.g., cancer of unidentified primary, or CUPs). 

Finally, our findings indicate missing opportunities regarding how the tumor ECM is being considered in pharmacological approaches. The tumor ECM structures, namely, the ECM-rich regions, are not typically targeted in cancer therapeutics; thus, even if cancer cells are removed, patients are left with extremely stiff and large fibronectin-rich structures in their lungs. Therefore, if left untreated, these structures could not only leave them with lifelong breathing difficulties but, since fibronectin is a chemoattractant and promotes tumor growth, could facilitate “re-invasion” of the tissue. Collectively, our results shed new light on the role of fibronectin in the reorganization and mechanical changes of the ECM during lung metastasis, and point to the connection between fibronectin secretion, tumor hypoxia, cell necrosis, and potentially LOX expression in the process of tumor development. As such, these interconnections suggest a new arena of signaling cascades that can be targeted against lung metastasis. 

## 5. Conclusions

This study aimed to characterize the extracellular matrix (ECM) of lung metastases from two different metastatic models: melanoma lung metastases (B16F10) and Lewis lung carcinoma metastases (LLC1). The results show that the tumor ECM is not as disorganized as previously thought, and that desmoplasia is not restricted to the tumor surroundings. Different ECM structures were found, likely formed by different mechanisms, and not by random deposition or degradation of the pre-metastatic ECM. The study linked ECM mechanical changes to changes in ECM composition, such as softening caused by basement membrane degradation, and identified fibronectin as a key ECM protein displaying deposition changes in every stage of lung metastases progression. Differences in the mechanical and invasion phenotypes were also observed between the two models, supporting the idea that different primary origins produce different tumor environments, while similarities in ECM composition could be attributed to an influence of the target organ environment on tumorigenesis. Overall, the findings in this article suggest that ECM mechanics can reflect underlying malignant mechanisms and evolve alongside tumor progression, and that understanding the differences in ECM between different tumors could aid in the development of new tumor microenvironment-targeting therapeutics.

## Data Availability

Data are available upon reasonable request.

## Supplementary Materials

The following supporting information can be downloaded at: https://www.mdpi.com/article/10.3390/cancers15082404/s1. Supplementary Figure S1. Correlation analysis between all measured variables in CAR and MEL metastases. (A,B) Pearson correlation matrix with Pearson correlation coefficients for CAR and MEL metastases, respectively. Green boxes indicate statistically significant correlations (*p* < 0.05). Relationship between ECM proteins (fibronectin, laminin, collagen I and IV) and (C) stiffness in CAR; (D) diameter in CAR; (E) stiffness in MEL and (F) diameter in MEL micro and macro metastases. Only significant and/or biologically relevant correlations were plotted. Supplementary Figure S2. Correlation analysis between all measured variables in NTD-treated MEL metastases. (A) Pearson correlation matrix with Pearson correlation coefficients for NTD-treated MEL metastases. Green boxes indicate statistically significant correlations (*p* < 0.05). Relationship between ECM proteins (fibronectin, laminin, collagen I and IV) and (B) tumor necrosis, measured by TUNEL+ areas; (C) ECM stiffness and (D) macrometastases’ diameter. Correlation analysis in MEL (treated (-) and non-treated (+)) between stiffness of the ECM-rich areas and (E) macrometastases diameter. *p*-values (*p*) obtained by computing the pearson correlation. Only significant and/or biologically relevant correlations were plotted. Supplementary Figure S3. Correlation analysis between all measured variables in NTD-treated MEL metastases. (A) Pearson correlation matrix with Pearson correlation coefficients for NTD-treated MEL metastases. Green boxes indicate statistically significant correlations (*p* < 0.05). Relationship between ECM proteins (fibronectin, laminin, collagen I and IV) and (B) tumor necrosis, measured by TUNEL+ areas; (C) ECM stiffness and (D) macrometastases’ diameter. Correlation analysis in MEL (treated (-) and non-treated (+)) between stiffness of the ECM-rich areas and (E) macrometastases diameter. *p*-values (*p*) obtained by computing the pearson correlation. Only significant and/or biologically relevant correlations were plotted.
