# Supplementary material for: Lung Micrometastases Display ECM Depletion and Softening While Macrometastases Are 30-Fold Stiffer and Enriched in Fibronectin
Source: Cancers (Basel). 2023 Apr 21;15(8):2404. doi: 10.3390/cancers15082404 (PMC10136590; doi:10.3390/cancers15082404)
Supplement: Supplementary file 1 [file cancers-15-02404-s001.zip › cancers-2318286-supplementary.pdf]

SUPPLEMENTARY MATERIAL

"Lung Micrometastases Display ECM Depletion and Softening While Macrometastases Are 30-Fold Stiffer and Enriched in Fibronectin"

Supplementary Figures and Figure legends

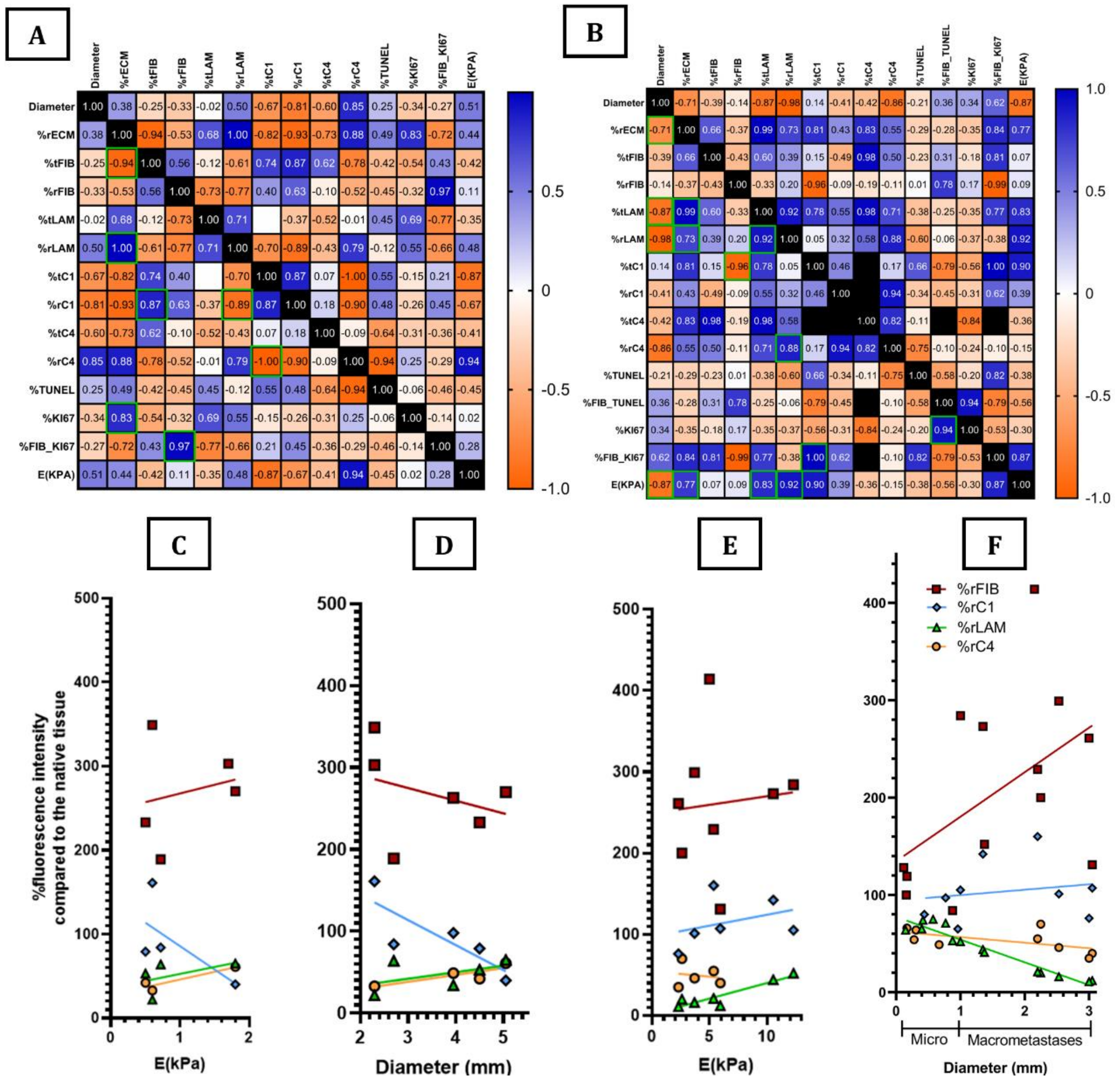

Supplementary Figure S1 - Correlation analysis between all measured variables in CAR and MEL metastases. (A-B) Pearson correlation matrix with Pearson correlation coefficients for CAR and MEL metastases, respectively. Green boxes indicate statistically significant correlations ($p<0.05$). Relationship between ECM proteins (fibronectin, laminin, collagen I and IV) and (C) stiffness in CAR; (D) diameter in CAR; (E) stiffness in MEL and (F) diameter in MEL micro and macro metastases. Only significant and/or biologically relevant correlations were plotted.

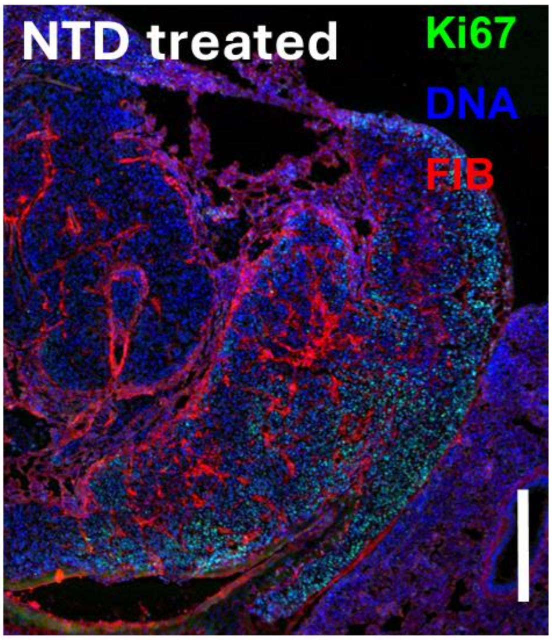

Supplementary Figure S2 – Representative image of tumor proliferation (Ki67) and fibronectin in MEL macrometastasis after NTD treatment. In blue, DNA staining (Nucblue), in green Ki67+ areas and in red, fibronectin. Scale bar = 400 μm.

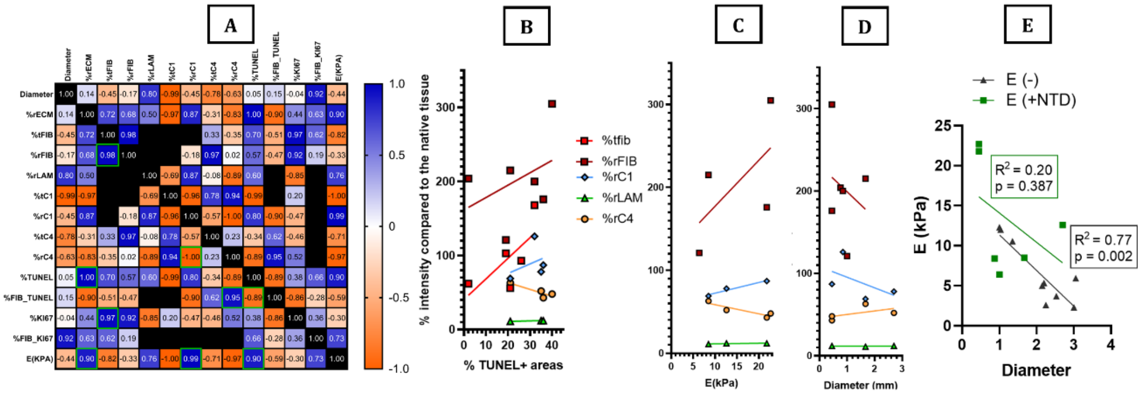

Supplementary Figure S3 - Correlation analysis between all measured variables in NTD-treated MEL metastases. (A) Pearson correlation matrix with Pearson correlation coefficients for NTD-treated MEL metastases. Green boxes indicate statistically significant correlations ($p<0.05$). Relationship between ECM proteins (fibronectin, laminin, collagen I and IV) and (B) tumor necrosis, measured by TUNEL+ areas; (C) ECM stiffness and (D) macrometastases' diameter. Correlation analysis in MEL (treated (-) and non-treated (+)) between stiffness of the ECM-rich areas and (E) macrometastases diameter. P-values (p) obtained by computing the pearson correlation. Only significant and/or biologically relevant correlations were plotted.
